# Supplementary material for: Glycoproteomic landscape and structural dynamics of TIM family immune checkpoints enabled by mucinase SmE
Source: Nat Commun. 2023 Oct 4;14:6169. doi: 10.1038/s41467-023-41756-y (PMC10550946; doi:10.1038/s41467-023-41756-y)
Supplement: Supplementary file 1 — Supplementary Information [file 41467_2023_41756_MOESM1_ESM.pdf]

Supplementary information for:

Glycoproteomic landscape and structural dynamics of TIM family immune checkpoints enabled by mucinase SmE

Joann Chongsaritsinsuk‡, Alexandra Steigmeyer‡, Keira E. Mahoney‡, Mia A. Rosenfeld‡, Taryn M. Lucas, Courtney M. Smith, Alice Li, Deniz Ince, Fiona L. Kearns, Alexandria S. Battison, Marie A. Hollenhorst, D. Judy Shon, Katherine H. Tiemeyer, Victor Attah, Catherine Kwon, Carolyn R. Bertozzi, Michael J. Ferracane, Mark A. Lemmon, Rommie E. Amaro, Stacy A. Malaker*

‡ These authors contributed equally to the manuscript.

*Correspondence should be addressed to S.A.M.
Email: stacy.malaker@yale.edu

This PDF file includes:

Supplementary Figures 1 to 20
Supplementary References

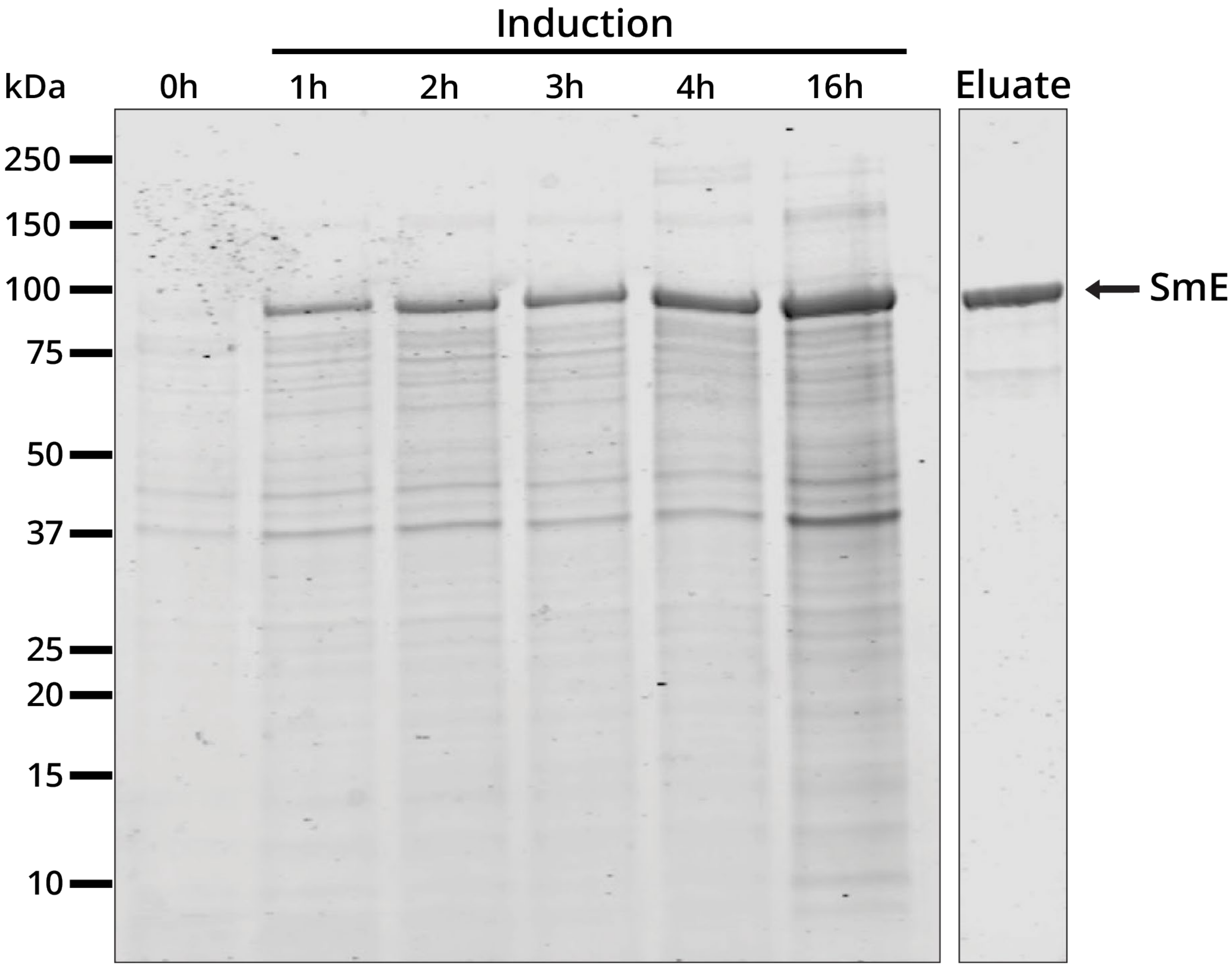

Supplementary Figure 1. Expression and purification of SmE. SmE was expressed overnight at 16 °C. SDS -PAGE gel was stained with Coomassie (Bulldog-Bio) and imaged using an Odyssey CLx Near-Infrared Fluorescence ImagingSystem (LI-COR Biosciences). SmE ran at the predicted molecular weight of 94 kDa, as indicated by the arrow.

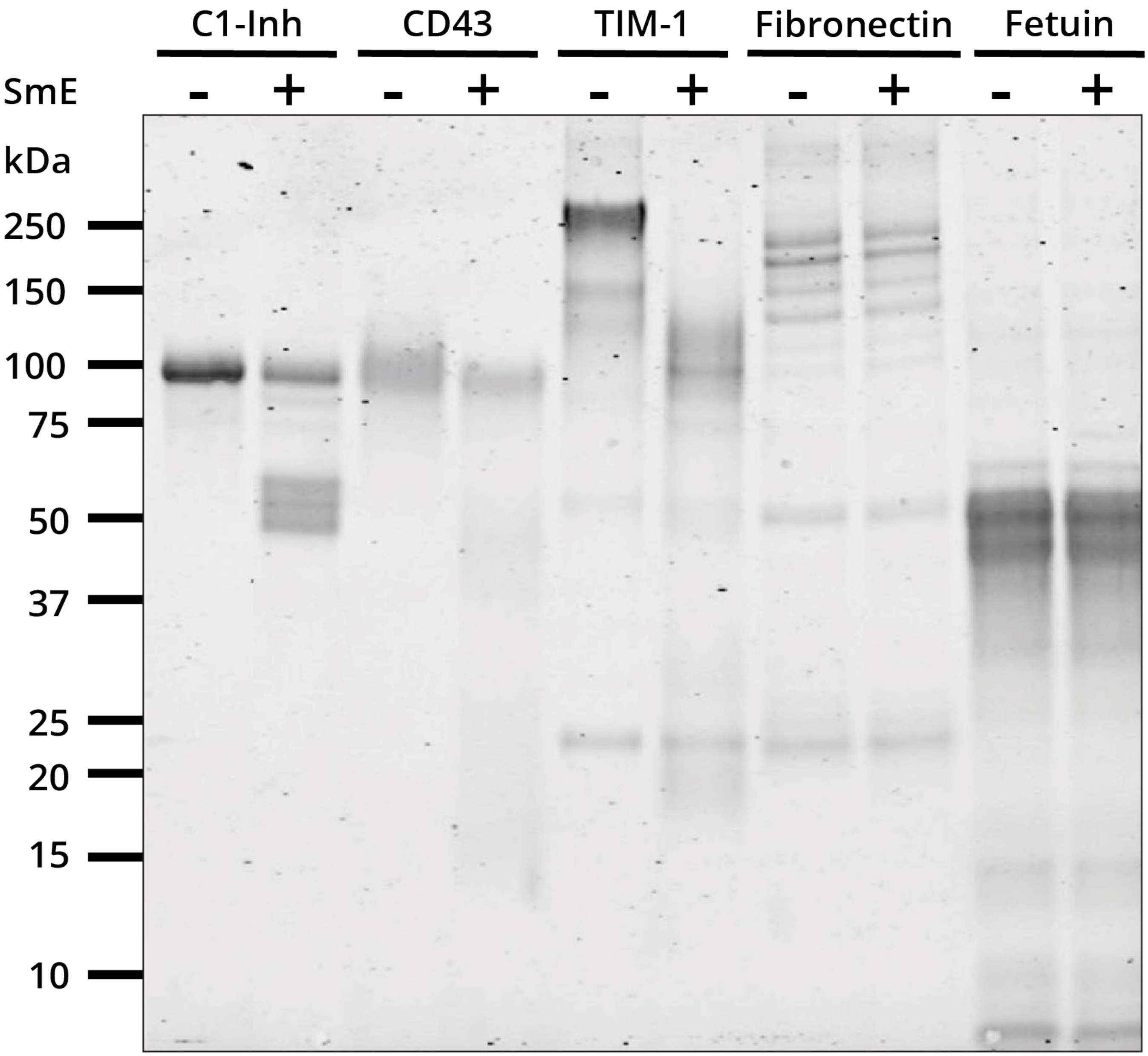

Supplementary Figure 2. SmE selectivity for mucin-domain glycoproteins. The recombinant proteins shown wereincubated with SmE at a 1:20 E:S ratio overnight at 37 °C and the digests were separated by SDS-PAGE. SDS-PAGE gel was stained with Coomassie (Bulldog-Bio) and visualized on an Odyssey CLx Near-Infrared Fluorescence Imaging System (LI-COR Biosciences). Limited digestion of CD43 is likely due to its expression in NS0 cells (*vide infra)*.

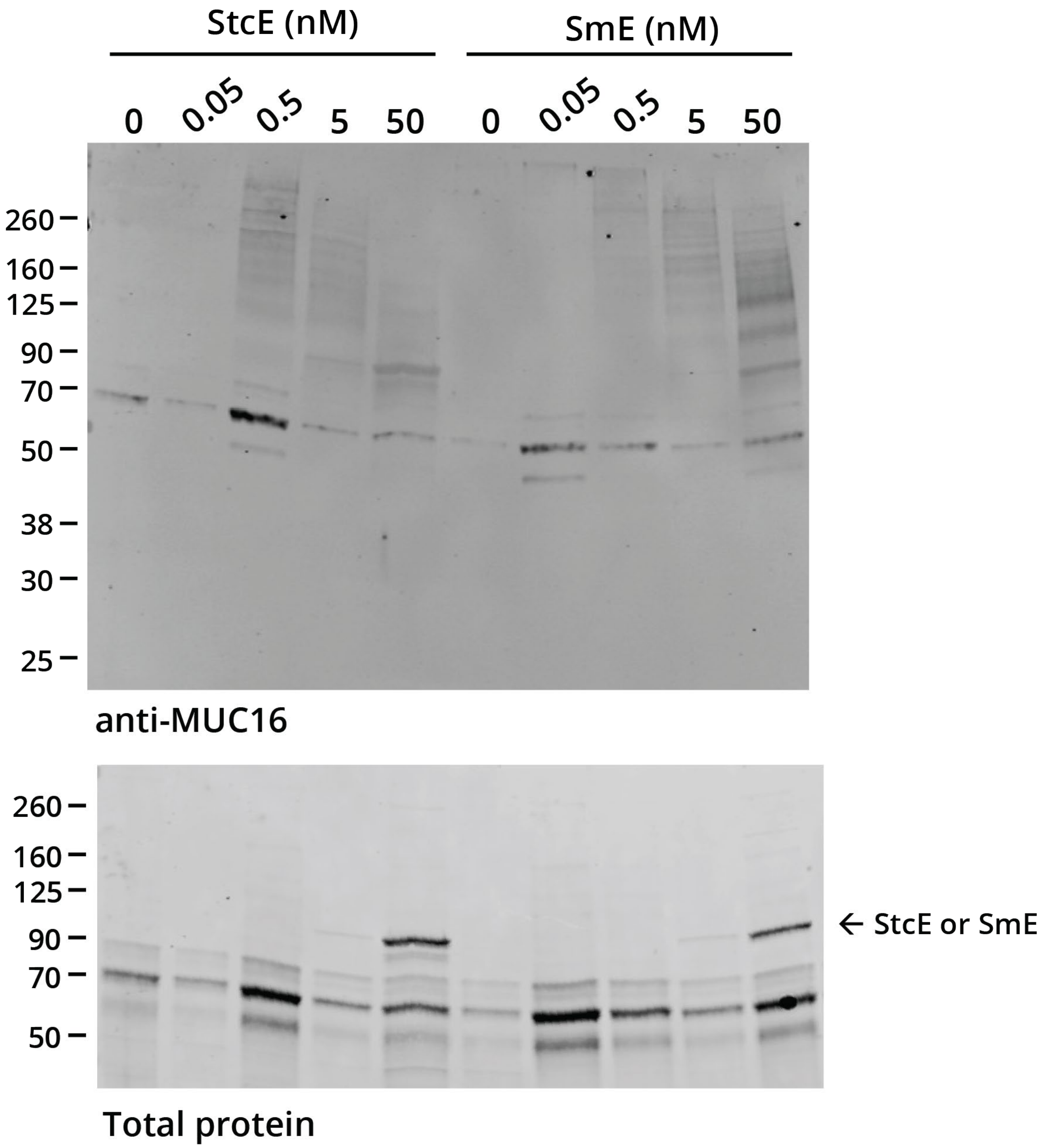

Supplementary Figure 3. MUC16 is released into cell supernatant after mucinase treatment. HeLa cells were treated with StcE (left) or SmE (right) at the noted concentrations for 60 min. Following treatment, the supernatant was transferred to 15 mL conical tubes containing 75 µL of EDTA to stop enzyme processing. The supernatants were then concentrated using 3 kDa MWCO spin filters and then diluted with 4X LDS sample buffer to a final concentration of 1X. The samples were then boiled at 95 °C for 5 min and loaded onto a 4-12% Bis Tris gel for separation by gel electrophoresis and probing for MUC16 via Western blot. Proteins were transferred to a nitrocellulose membrane using the Trans-Blot Turbo Transfer System (Bio-Rad) at a constant 2.5 A for 15 min. Total protein was quantified using REVERT stain before primary antibody incubation overnight at 4 °C. An IR800 dye labeled secondary antibody was used according to manufacturer's instructions for visualization on a Licor Odyssey instrument.

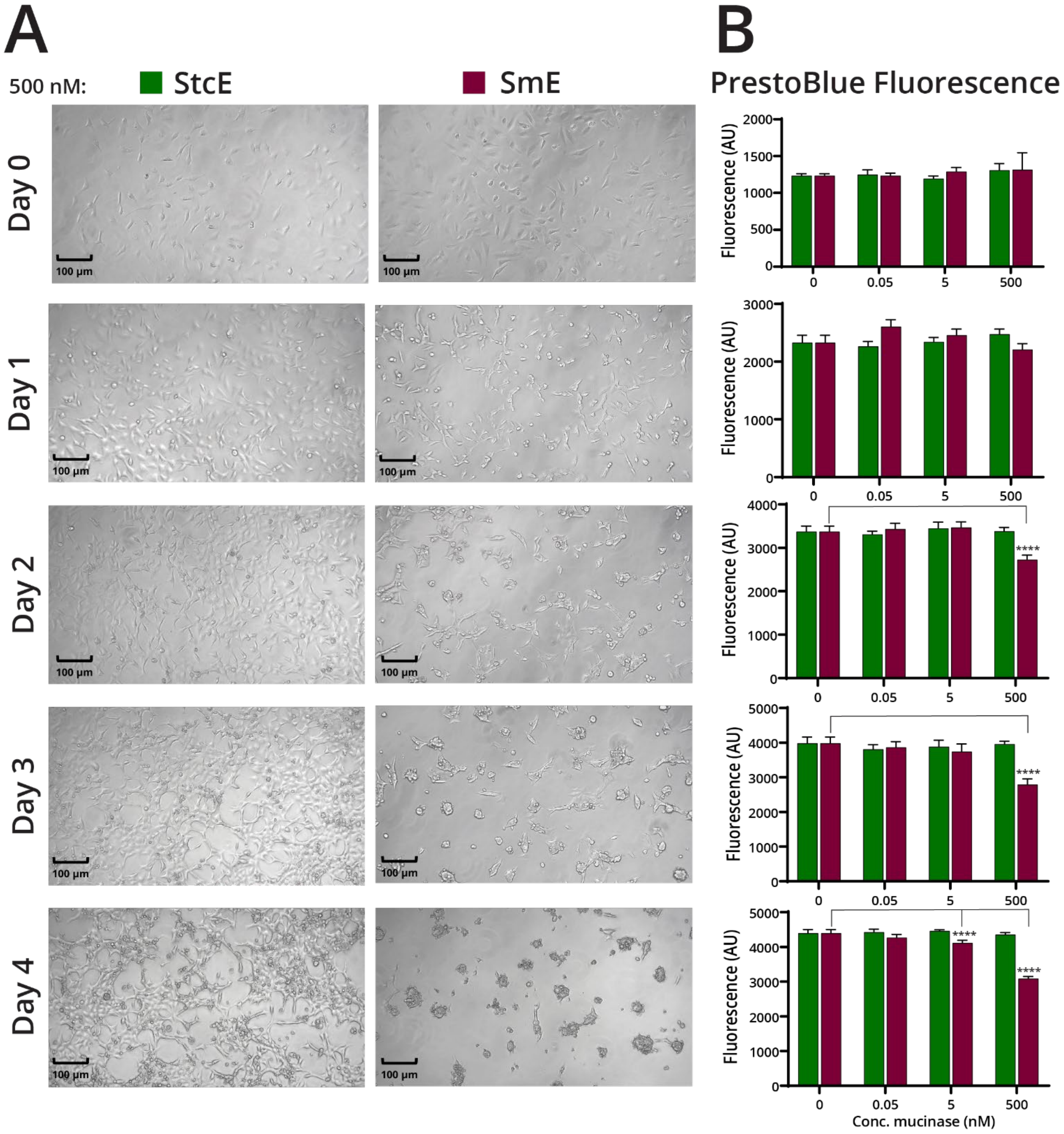

Supplementary Figure 4. SmE treatment was nontoxic to HeLa cells at moderate concentrations and/or durations. (A) Live cell microscopic images of HeLa cells treated with 500 nM StcE or SmE, taken every 24 hours for a total of 4 days. (B) Cellular viability was measured using a resorufin-based dye (PrestoBlue, Thermo Fisher Scientific), at 0, 0.05, 5 and 500 nM StcE or SmE treatment over 4 days. Green: StcE, maroon: SmE. Statistical significance was determined using the two-way ANOVA analysis in Graphpad PRISM software and is reported with respectto the no mucinase control condition. **** indicates a p value <0.0001. Scale bar = 100 µm.

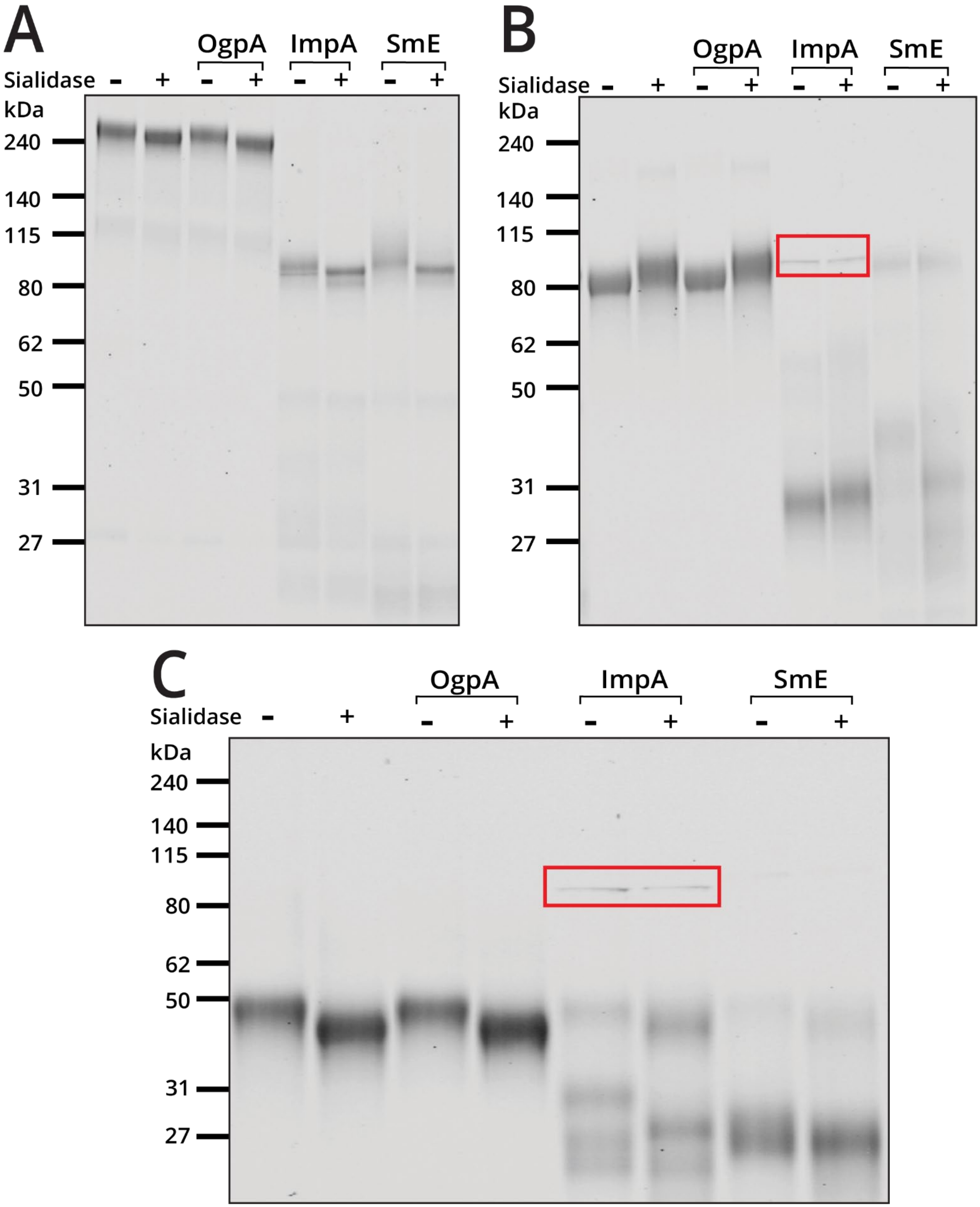

Supplementary Figure 5. **Benchmarking OgpA, ImpA, and SmE activity on TIM proteins.** Recombinant pr oteins (A) TIM-1, (B) TIM-4 and (C) TIM-3 were reacted with O-glycoproteases with and without sialidase over night at 37 °C. For SmE digestion, we employed a 1:10 E:S ratio, while for OgpA and ImpA, manufacturer's recommended conditions were used. All digests were separated via SDS-PAGE and stained with Coomassie (Bulldog-Bio). Gels were imaged on an Odyssey CLx Near-Infrared Fluorescence Imaging System (LI-COR Biosc iences). ImpA is denoted by the red box.

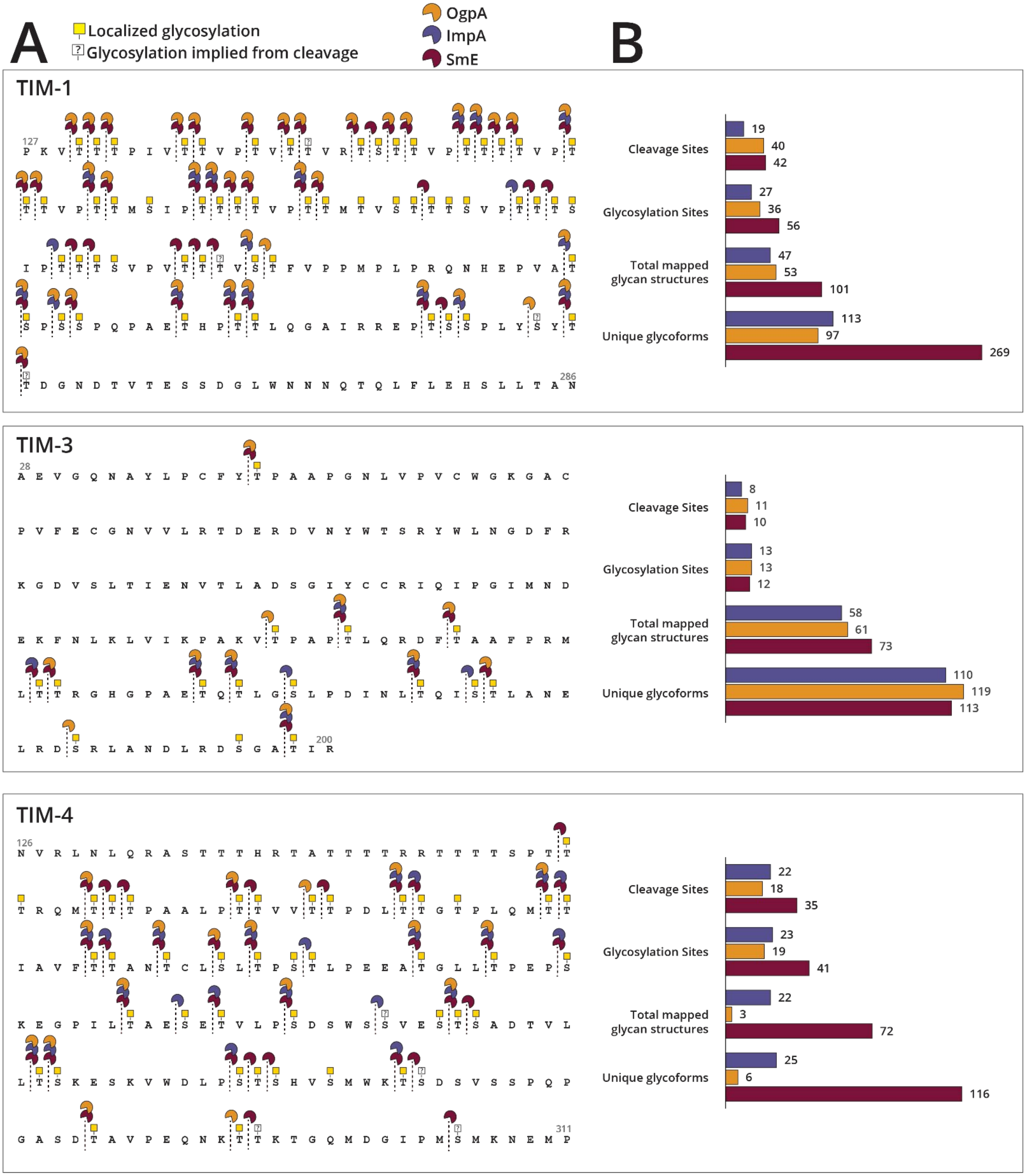
A
Localized glycosylation
Glycosylation implied from cleavage
OgpA
ImpA
SmE
B
TIM-1
TIM-3
TIM-4
Cleavage Sites
Glycosylation Sites
Total mapped glycan structures
Unique glycoforms
19
40
42
27
36
56
47
53
101
113
97
269
8
11
10
13
13
12
58
61
73
110
119
113
22
18
35
23
19
41
22
3
72
25
6
116

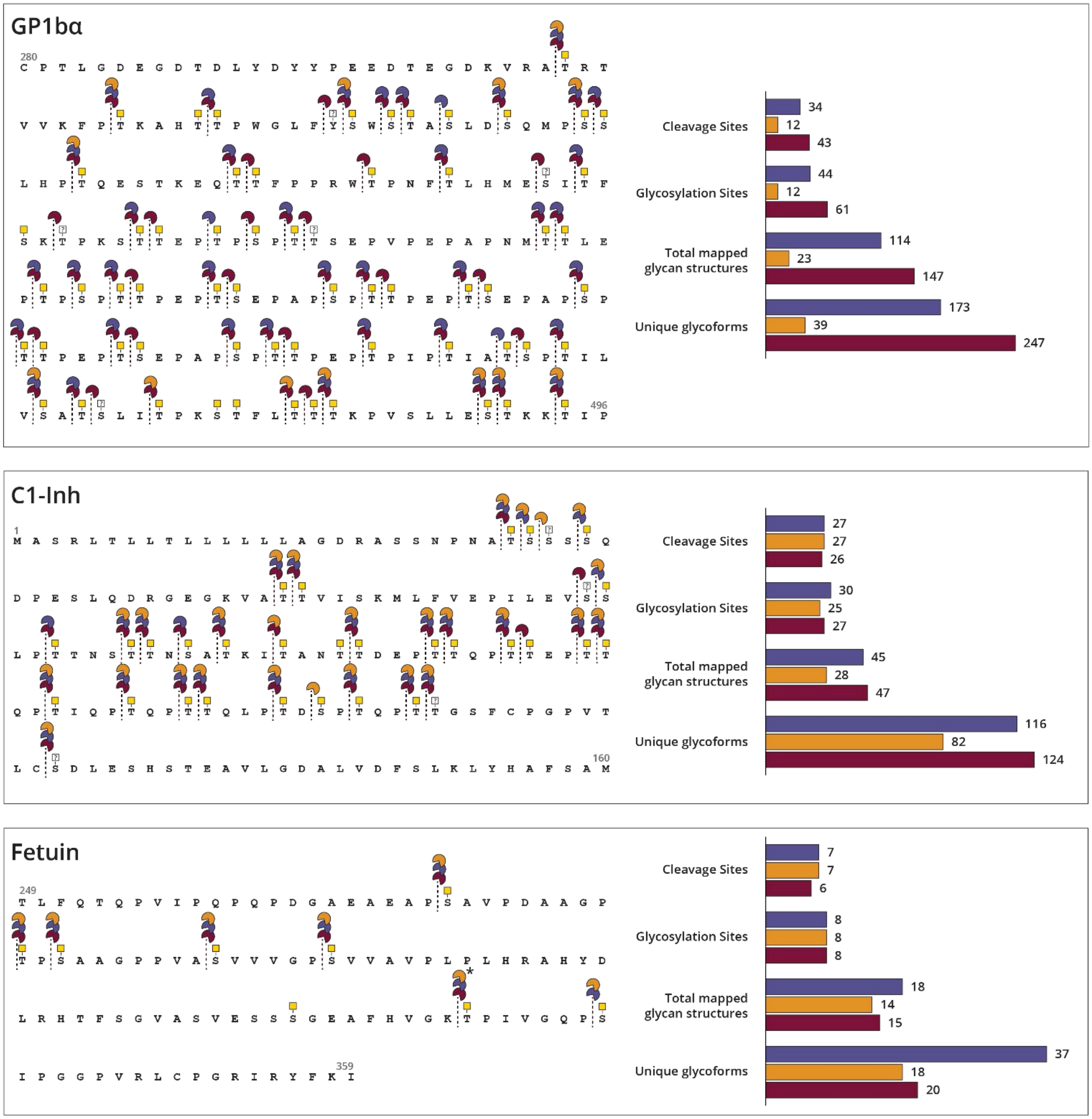

Supplementary Figure 6. Total cleavage events. (A) Cleavage maps depict observed glycosites and cleavag e sites of each enzyme from MS analysis. (B) Graphical interpretation of individual glycoprotein cleavage sites, glycosites, and glycoforms identified by treatment with each enzyme. Red: SmE, blue: ImpA, yellow: OgpA. A sterisk in fetuin cleavage map indicates cleavage attributable to either mucinase or co-enzyme (trypsin); white squares withquestion marks indicate implied glycosites with observed cleavage.

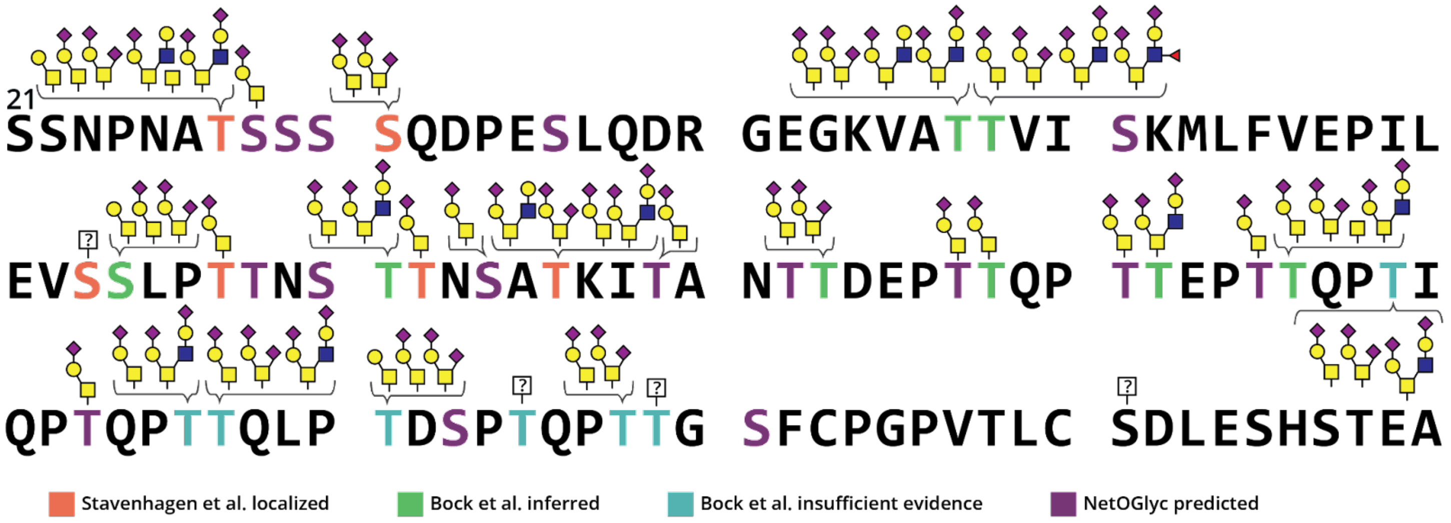

Supplementary Figure 7. C1-Inh glycoproteomic landscape. C1-Inh isolated from human plasma was subjeted to digestion with SmE and trypsin followed by MS analysis and manual data validation. Depicted are r esidues 21-140 which comprise the C1-Inh mucin domain. Glycans depicted in brackets were detected on the a ssociated residues. The colored residues were either detected (orange; Stavenhagen et al.),1 inferred (green; B ock et al.),2 hinted at without sufficient evidence (blue; Bock et al.),2 or predicted by NetOGlyc 4.0 (purple).

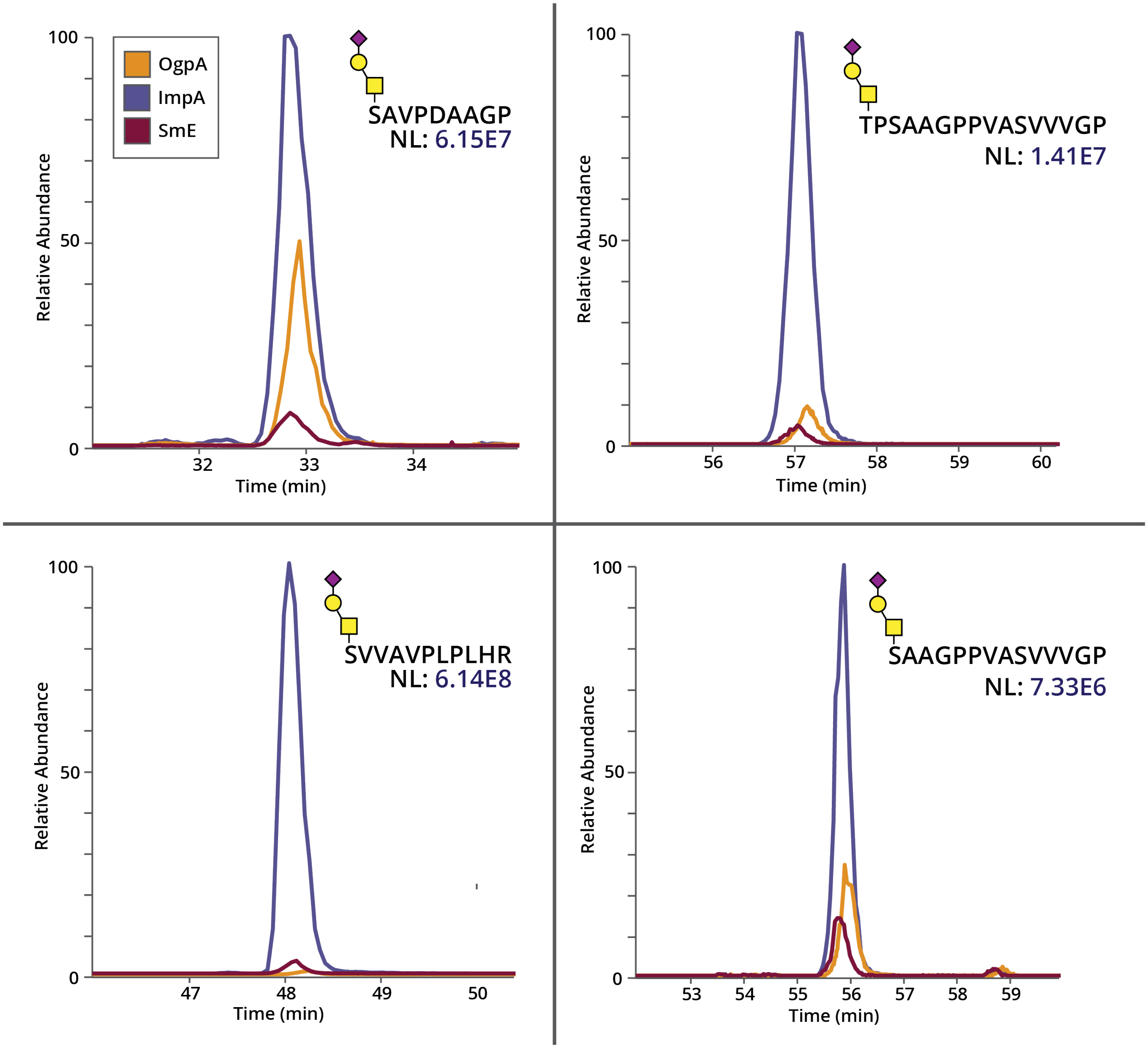

Supplementary Figure 8. Extracted ion chromatograms of cleaved glycopeptides from fetuin. Using Th ermo Xcalibur, extracted ion chromatograms (XICs) were generated for four glycopeptides from the OgpA, I mpA, and SmE digest of fetuin. The XICs of specific glycopeptides were normalized to indicate relative abu ndance in each analysis. Yellow: OgpA, blue: ImpA, red: SmE.

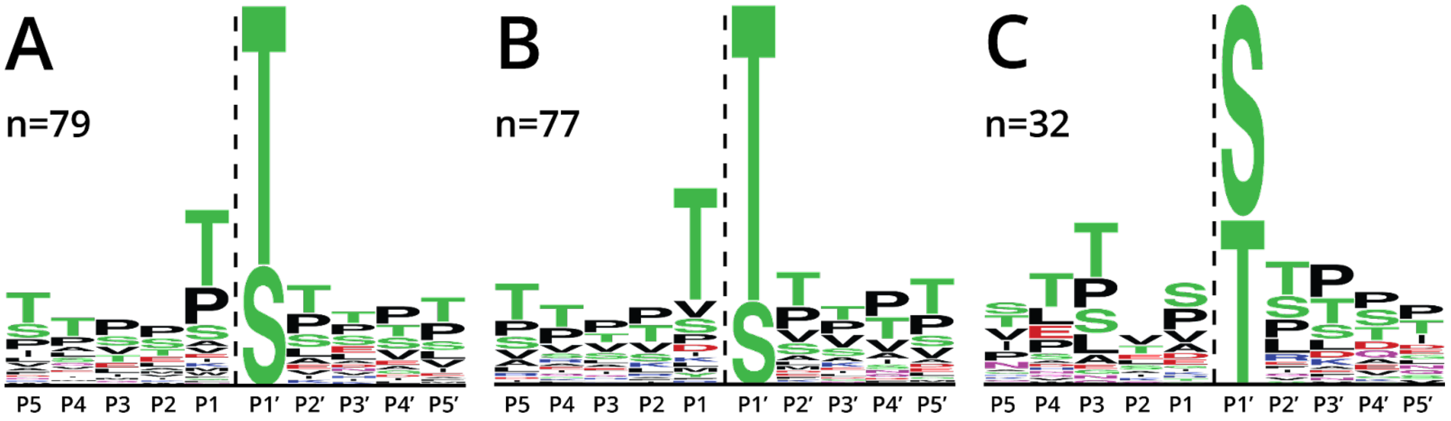

Figure S9. Anti-logos demonstrate missed cleavage events and less preferred P1/P1' residues. Using cleavage maps from Figure S5, all sites without observed cleavage were loaded into weblogo.berkeley.edu. OgpA (A) and ImpA (B) demonstrated less efficiency with Thr in the P1 position. SmE (C) uniquely showed lower preference for Ser in the P1' position.

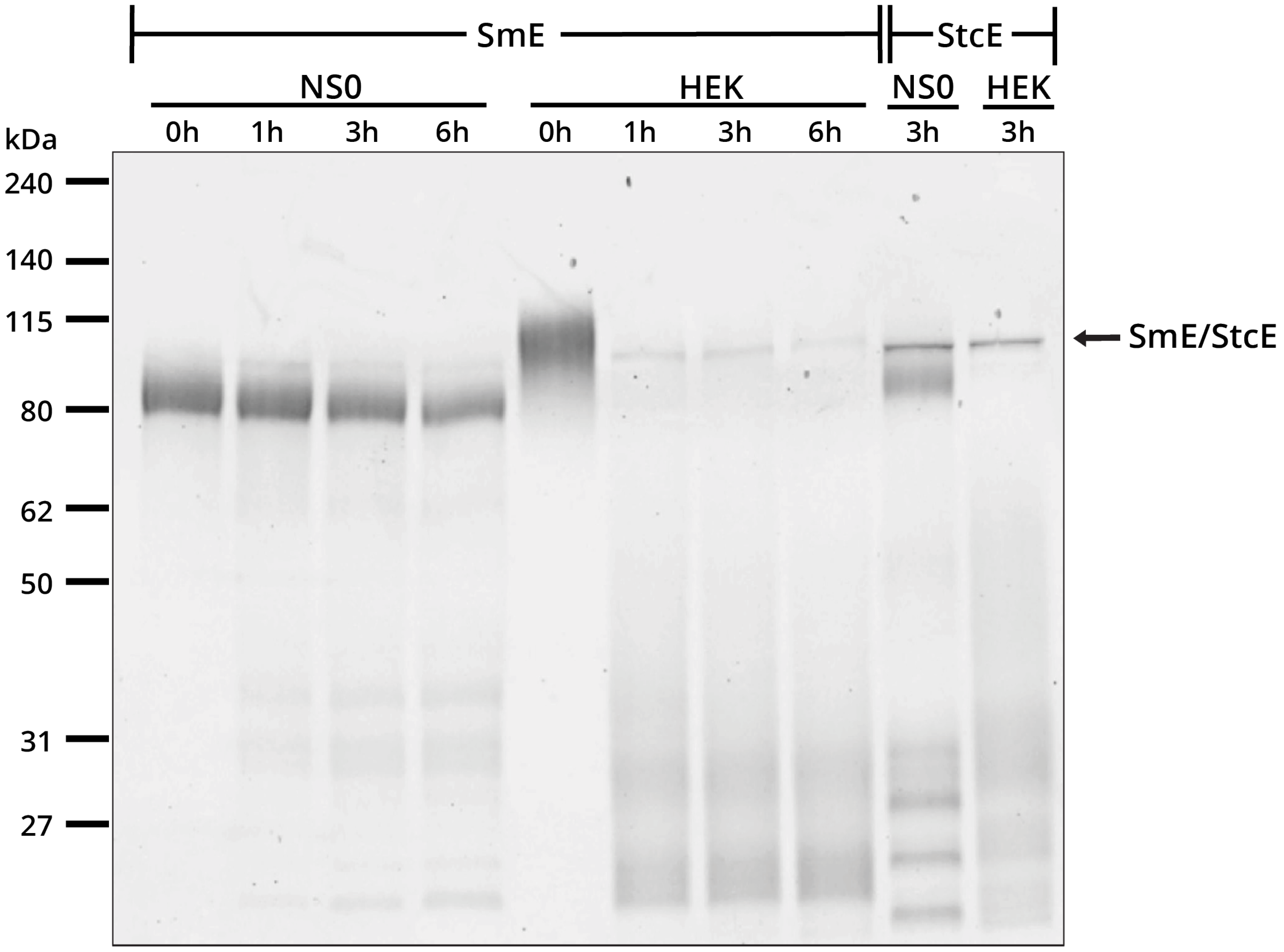

Supplementary Figure 10. Comparison of SmE activity on recombinant mouse (NS0) or human (HEK293)-derived TIM-1. Recombinant human TIM-1 expressed in NS0 or HEK293 cells were reacted with SmE at a 1:10 E:S ratio for 1, 3, or 6 hours at 37 °C and StcE at a 1:10 E:S ratio for 3 hours at 37 °C. All digests were separated by SDS-PAGE and Coomassie stained (Bulldog-Bio). Gel was visualized on an Odyssey CLx Near-Infrared Fluorescence Imaging System (LI-COR Biosciences).

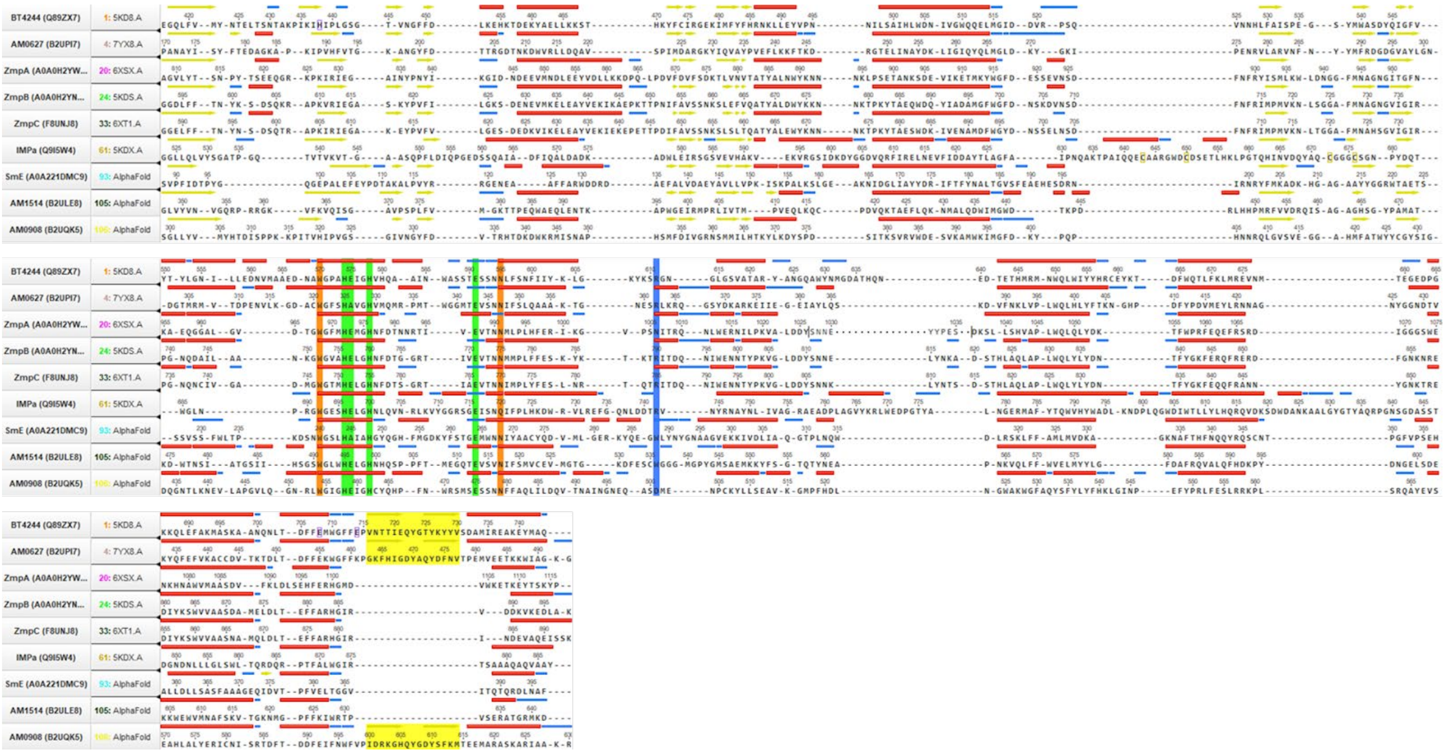

Supplementary Figure 11. Sequence alignment of the catalytic PF13402 domains found in cha racterized O-glycoproteases. The X-ray crystal structures of BT4244, AM0627, ZmpA, ZmpB, ZmpC, and ImpA as well as the AlphaFold-predicted structures of SmE, AM1514, and AM0908 were structurally overlaid using the conserved zinc-binding and catalytic residues (green). The structural overlays were then used to generate the above sequence alignments, highlighting key secondary structures: alpha helices (red), beta she ets (underlined yellow), and turns (underlined blue). In addition to the catalytic core residues (green), we have highlighted conserved (orange) and semiconserved (highlighted blue) residues involved in recognizing P1’ glycans, as well as residues in a semiconserved beta-hairpin (highlighted yellow) that can potentially recognize P1 glycans. Notably, SmE has neither the semiconserved Arg residue nor the semiconserved beta-hairpin.

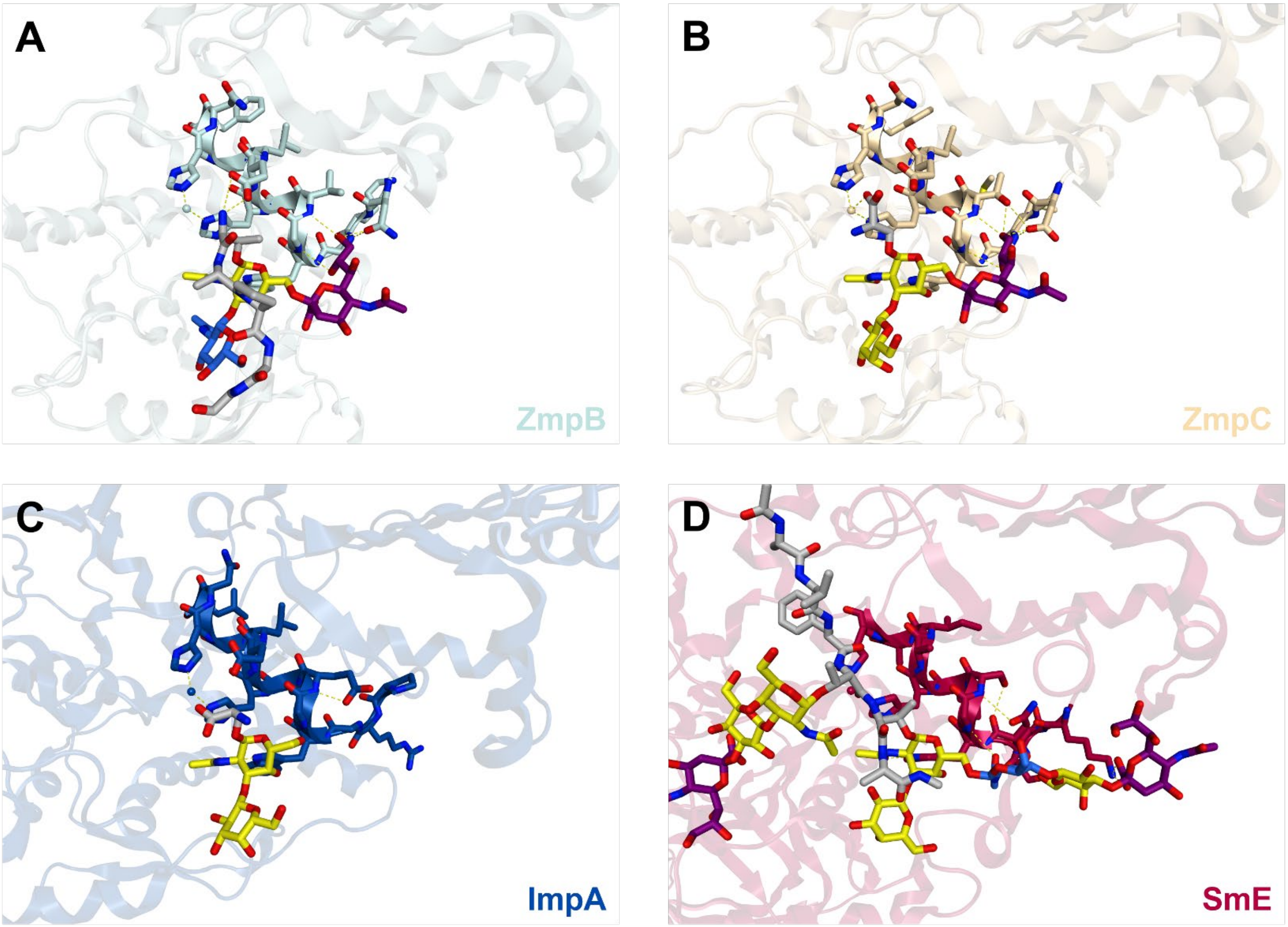

Supplementary Figure 12. Ligand-bound crystal structures of O-glycoproteases. Structures of ligand-bound catalytic helices of (A) ZmpB, (B) ZmpC, and (C) ImpA as well as the modeled structure of (D) SmE. Both ZmpB and ZmpC form specific contacts (yellow dashes) between residues near the terminus of the catalytic helix (colored sticks) and the branching sialic acid residue (purple sticks) found in the ligand at P1'. The ligand in ImpA does not contain sialic acid; analogous interactions can likely form, given the similar length of its catalytic helix, imparting ImpA with the ability to accommodate branched ligands. In the modeled structure, SmE forms contacts with the GalNAc residue (yellow sticks) and additional contacts with the sialic acid residue (purple sticks) of the glycan, which may explain its ability to accommodate larger branched glycans at P1'.

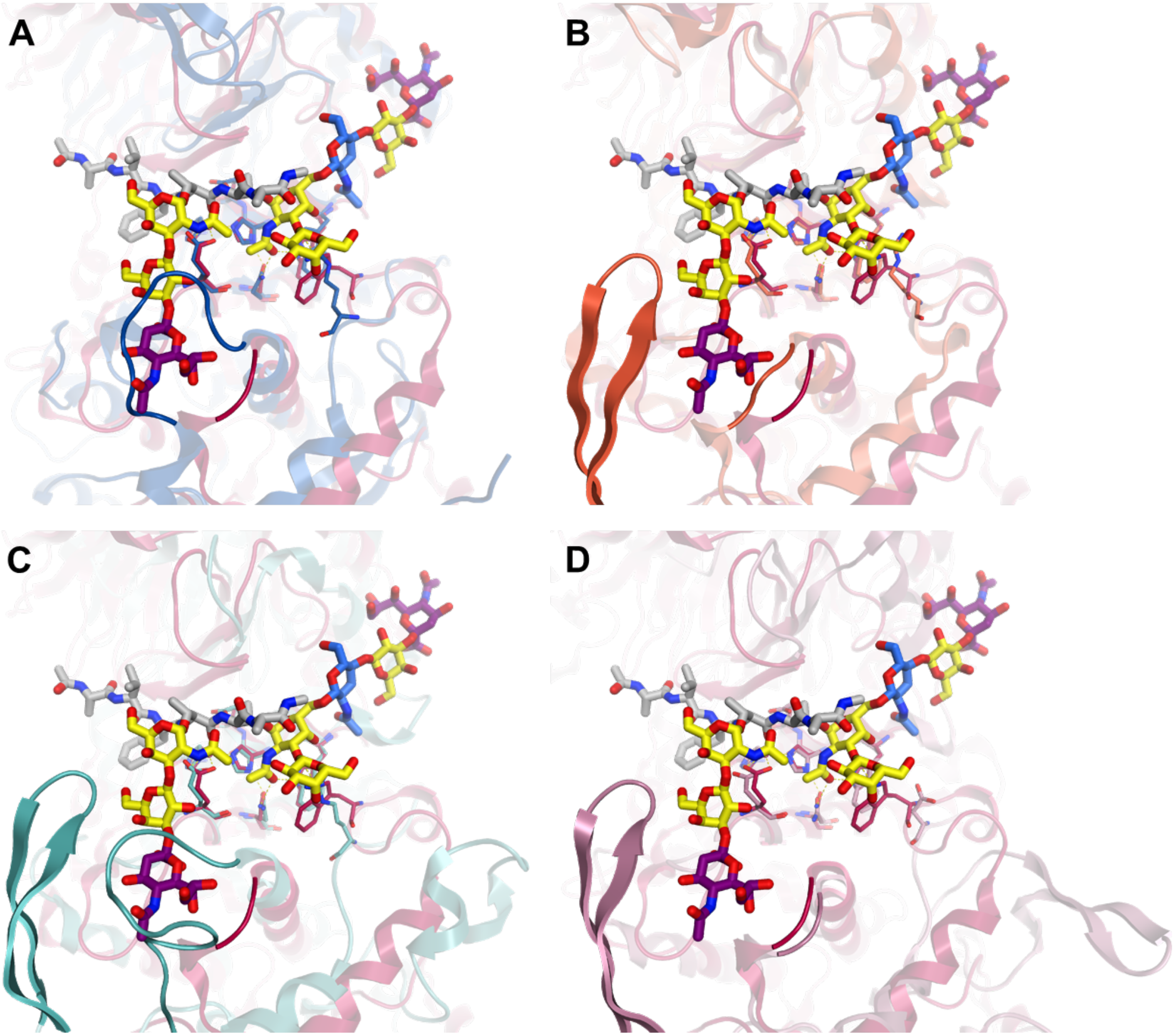

Supplementary Figure 13. Structural alignments of O-glycoproteases. Overlays of the docked SmE-glycop eptide complex (red) with the crystal structures of (A) ImpA (blue), (B) AM0627 (orange), and (C) BT4244 (mint) as well as the AlphaFold predicted structure of (D) AM0908 (pink). The residues (thin sticks) of the catalytic core and the conserved residues that bind the P1' glycan are shown with the docked ligand (thick sticks). The loops and beta hairpins that form the steric environment around the P1 glycan are darkened for emphasis. (A) The loop in SmE is predicted to be short, which could explain why SmE can sterically accommodate and act on substrates with glycosylation at P1; the loop of ImpA is long and likely prevents such activity. (B) AM0627 has a short loop that can accommodate different glycans at P1. (C) The loop of BT4244 is much larger, and this difference could resolve discrepancies reported across the literature and may explain why BT4244 showed preference for engineered substrates bearing the smaller Tn-antigen.
(D) AM0908 is predicted to have a short loop similar to AM0627; despite this potential similarity, the two enzymes display different preference for P1 glycosylation, which cannot be explained by this reasoning and may be the result of subtler differences in their sequences.

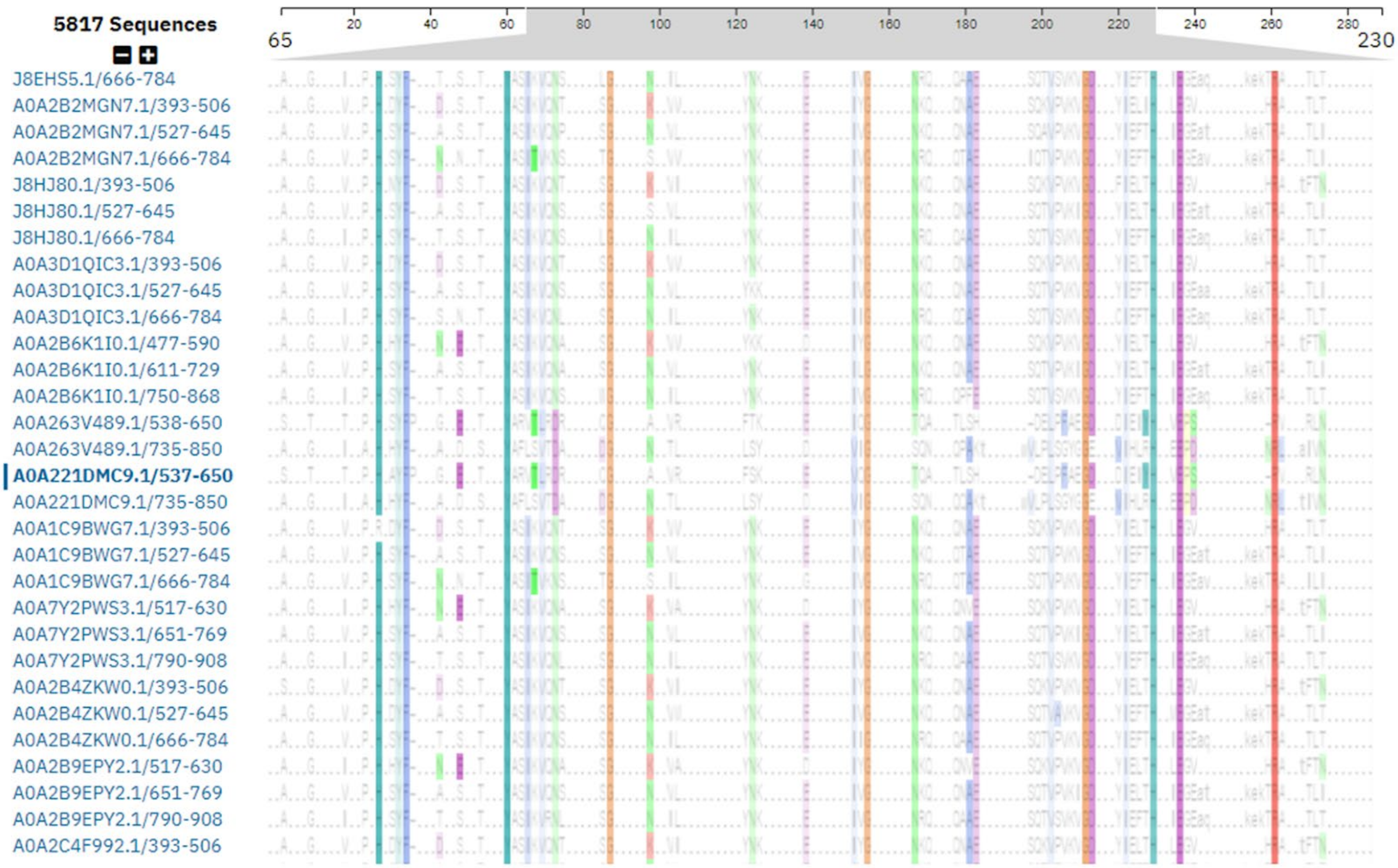

Supplementary Figure 14. **Sequence alignment of PF03272 modules.** Conservation across the 5,817 mucin-binding modules (PF03272) found in proteins, including SmE (A0A221DMC9.1/537-650), listed in UniP rot. Degree of conservation is shown using clustal2 coloring, highlighting two conserved motifs (HxxFxxxxY and HxExxR) found in loops clustered together to form a single binding pocket in the AlphaFold structure of SmE. Full results areavailable at https://www.ebi.ac.uk/interpro/entry/pfam/PF03272/entry_alignments/?type=uniport.

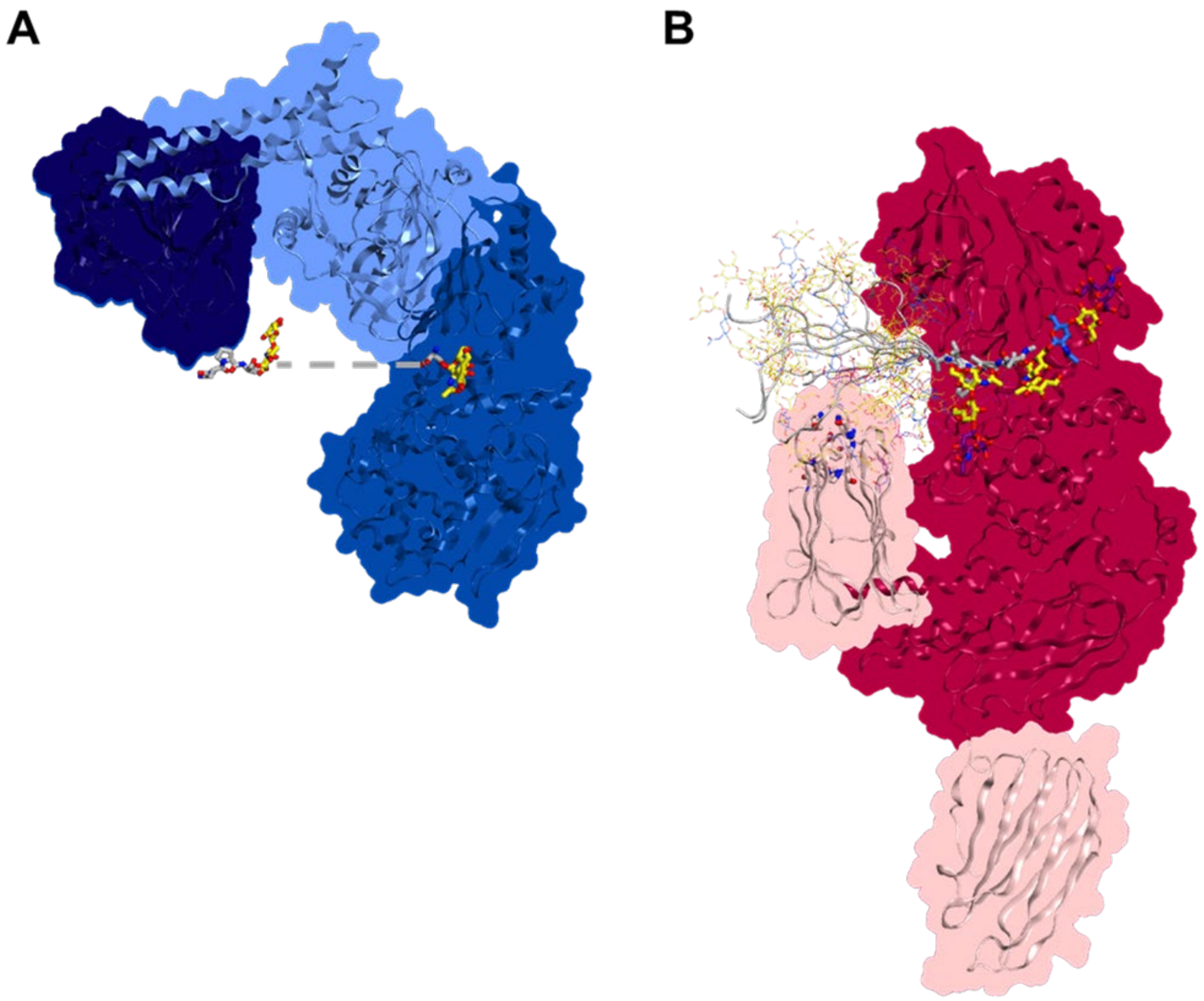

Supplementary Figure 15. Potential cooperativity of ligand binding in ImpA and SmE. (A) Overlay of cryst al structures of ImpA with crystallized ligands in the catalytic and accessory domains. The ligand (thick sticks) crystallized in the catalytic domain (blue loops and shaded surface) as well as the ligand (thick sticks) in the accessory N-terminal domain (dark blue loops and shaded surface) are small fragments that likely reflect the orientation of larger mucin-like substrates (dashed line), highlighting potential cooperativity between the different domains in ImpA. (B) Grafted TIM-4 fragments overlaid with the modeled structure of SmE. The catalytic domain (red loops and shaded surface) and the two mucin-binding modules (light pink loops and shaded surface) are shown, including the conserved motifs (HxxFxxxxY and HxExxR, thick sticks) in the loops of the mucin-binding module adjacent to the catalytic site. In addition, the amino acid and glycan residues of the original docked glycopeptide (thick sticks) is displayed, grafted together with ten different TIM-4 fragments. Only the peptide backbone (gray ribbon) and glycans (thin sticks) of these fragments have been highlighted for clarity. Some glycans were found to flank the conserved residues of the mucin-binding module, suggesting that the catalytic and accessory domains ofSmE may cooperatively recognize and cleave mucin-like substrates.

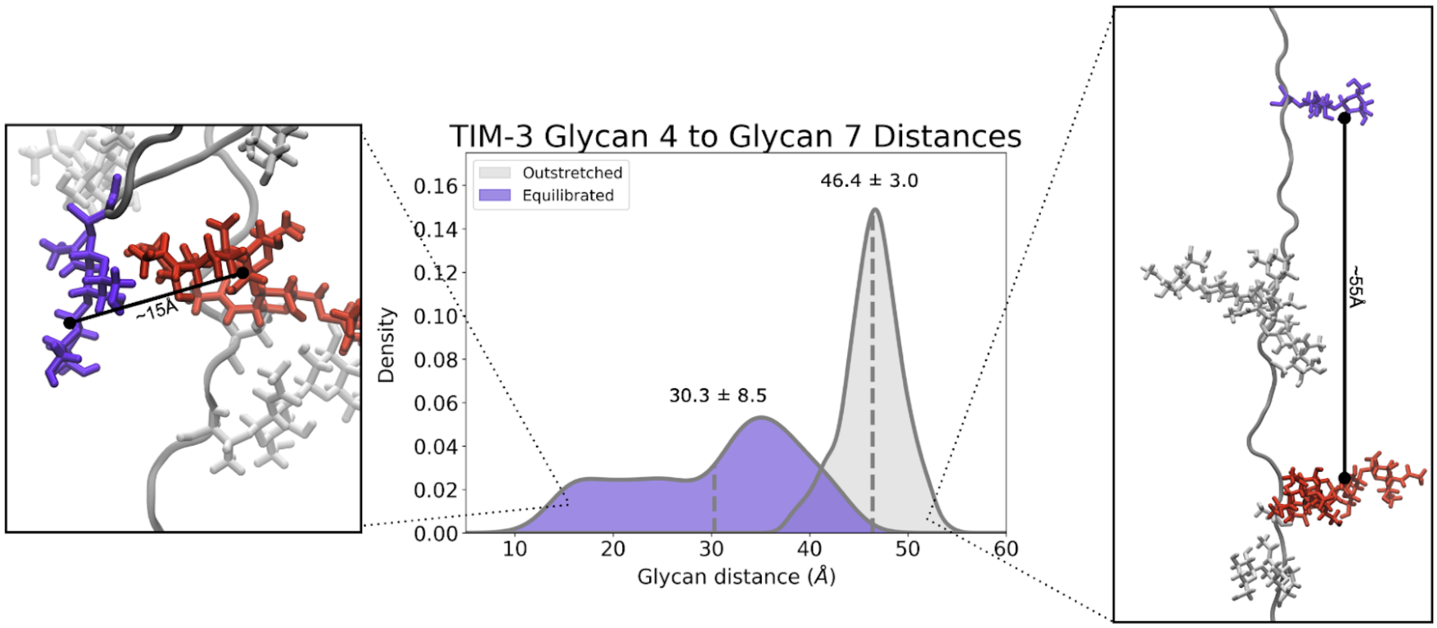

Supplementary Figure 16. **Histogram detailing distance between glycans G4 (site T162) and G7 (site T145) inoutstretched versus equilibrated conformation, measured throughout molecular dynamics simulation**. Left panel is a frame taken from an equilibrated conformation with a glycan distance of approximately 15 Å between G4 and G7. Right panel is a frame taken from an outstretched/not fully equilibrated conformation with a glycan distance of approximately 55 Å between G4 and G7.

179 TGTPLQMTTIAVFT|TANTCLSLTPSTLP 206

Glycan	Occupancy
H1N1A1	19.6%
H1N1	18.4%
N1	15.7%
n/a	12.9%
H1N1A2	12.0%
H2N2	9.8%
H1N2	3.5%
H2N2A1	3.3%
H1N1F1	2.9%
H1N2A1	1.1%
H2N2A2	0.6%
N2	0.1%

Glycan	Occupancy
H2N2A1	43.0%
N1	26.7%
H2N2	15.3%
H1N1	8.2%
H2N2F1	1.9%
H1N2	1.4%
H1N1A2	1.4%
H1N1A1	1.1%
H1N2A1	0.8%

Supplementary Figure 17. TIM-4 T192 and T193 glycan abundances. The relative abundance of different glycans branching from T192 and T193 of TIM-4, as revealed through digestion with SmE and subsequent MS analysis. The H1N1A1 and H2N2A1 glycans were the most abundant species at these positions, and were incorporated into the ligand used in docking experiments with SmE.

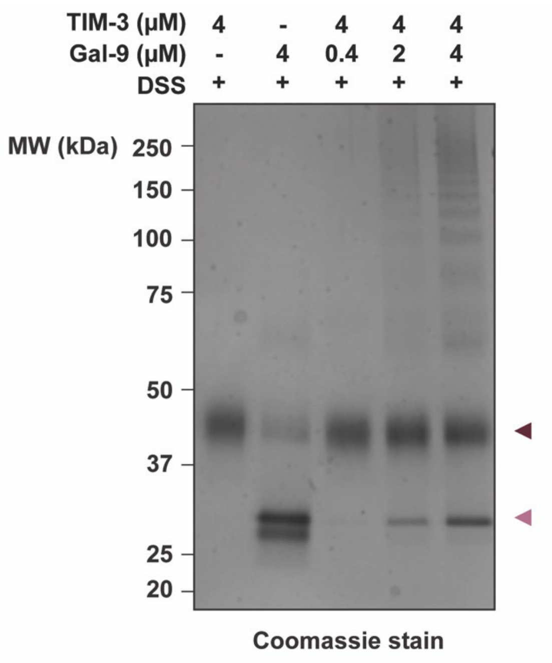

Supplementary Figure 18. Gal-9 crosslinking of TIM-3. Purified soluble TIM-3 extracellular region (TIM-3; 4 μM) was incubated with ssGal9WT (4, 2, or 0.4 μM) with or without the crosslinking reagent disuccinimidyl suberate (DSS; 300 μM). Samples were run on SDS-PAGE and analyzed by Coomassie blue staining. The maroon arrow indicates band corresponding to monomeric TIM-3, and the pink arrow indicates band corresponding to monomeric Gal-9. Higher molecular weight species are seen when DSS and Gal-9 are incubated with TIM-3.

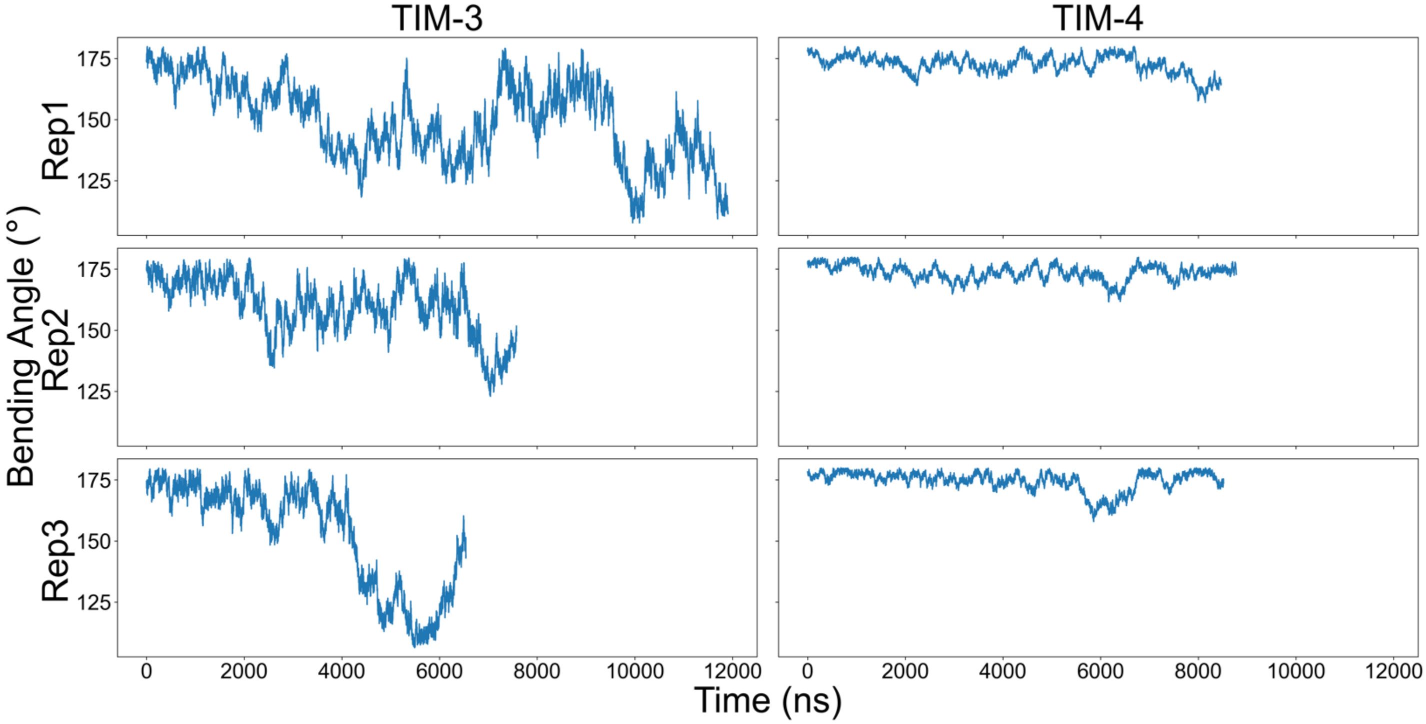

Supplementary Figure 19. Time-series analyses of mucin domain bending angles performed on a per-replica basis. Even on a per replica basis, TIM-3 models demonstrate marked variability in bending angle, while TIM-4 models dramatically decreased degrees of angle bending.

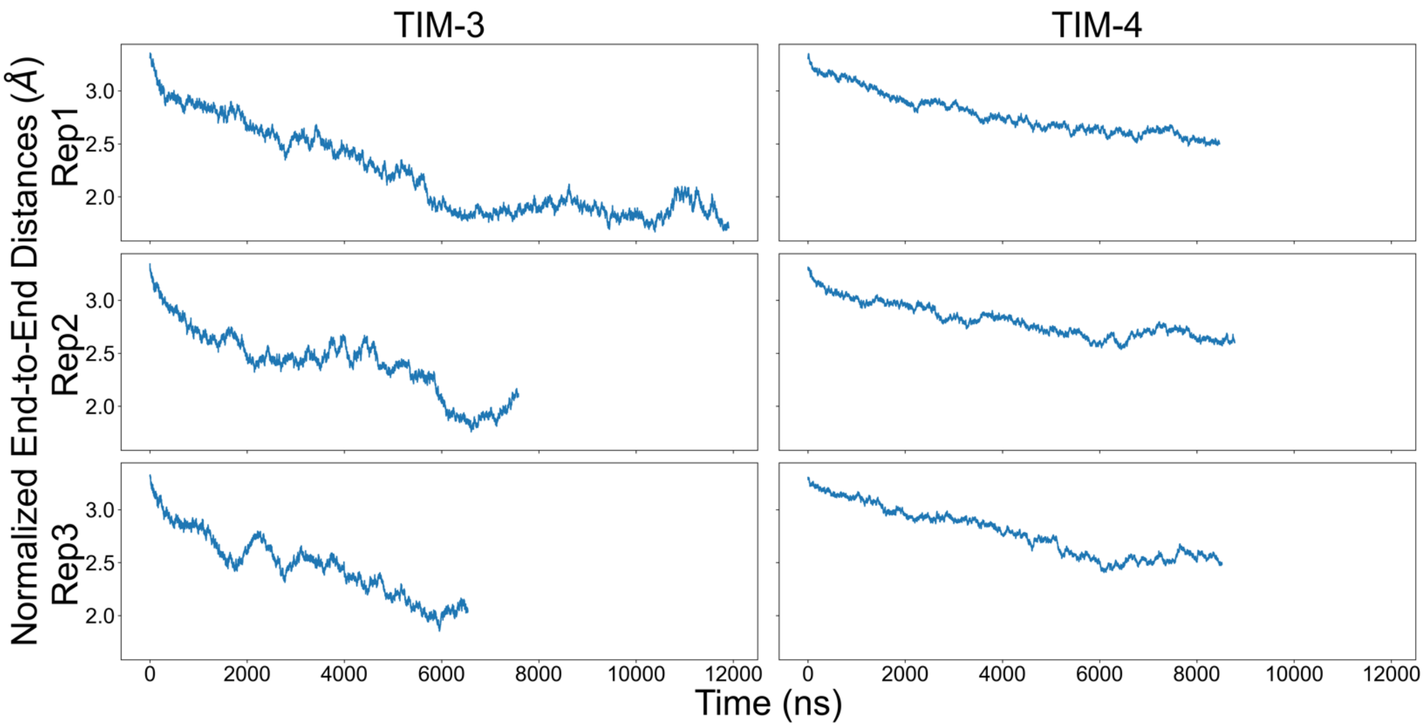

Supplementary Figure 20. Time-series analysis of mucin domain normalized end-to-end distances on a per-replica basis. Even on a per-replica basis, TIM-3 demonstrates a much lower normalized end-to-end distance than TIM-4.

Supplementary Notes

Limitations of SmE for glycoproteomic analyses

Although SmE clearly exhibited benefits for mucin glycoprotein analysis, we observed certain limitations associated with its use. Relative to its performance in mucin domains, the efficiency of SmE was greatly diminished when used on non-mucin glycoproteins, such as fetuin (Supplementary Figure 6; SI Table 6). Analysis by MS revealed that SmE was able to cleave non-mucin glycoproteins to a limited extent; however, the abundance of fetuin glycopeptides resulting from SmE digestion was significantly reduced when compared to those of ImpA, and in some cases, OgpA (Supplementary Figure 8). These observations, along with the digestion assay in Supplementary Figure 2, support the notion that SmE is a mucin-selective O-glycoprotease. While SmE greatly enhanced sequence coverage and depth, some glycosylation was only localized through the use of OgpA and ImpA (Figure 2D). For this reason, we recommend a complementary, multi-enzyme approach to fully elucidate the glycoproteomic landscape of mucin-domain glycoproteins. Additionally, since ImpA was previously reported to have P1 selectivity, we generated "anti-logos" to determine surrounding residues that were unfavorable for cleavage. Here, we considered the total cleavage maps depicted in Supplementary Figure 6, and whenever an enzyme did not cleave at an observed cleavage site, we took the surrounding amino acids and generated a logo. Interestingly, we found that SmE has a lower cleavage efficiency at Ser residues (Supplementary Figure 9), which may be an important consideration for digestion of mucin domains bearing high levels of Ser residues. Finally, we observed that SmE exhibited reduced proteolytic activity on recombinant proteins expressed in murine-derived cell lines, as exemplified by (a) the digestion of TIM-1 from NS0 and HEK cells over the course of six hours (Supplementary Figure 10) and (b) limited digestion of CD43 derived from NS0 cells (Supplementary Figure 2).

Molecular modeling identifies potential secondary mucin binding in SmE

Multidomain mucinases are hypothesized to arrange their noncatalytic domains into an architecture that enables specific recognition of secondary sites along the linear bottle-brush of mucins,[3,4] and recombinant StcE lacking one of these noncatalytic domains showed reduced activity on mucin substrates.[5,6] Interestingly, the related metalloprotease MMP-1 (collagenase) required an accessory domain to bind and cleave the linear triple helix of collagen;[7] structural and functional studies revealed the importance of cooperativity between this enzyme's catalytic and accessory domains as well as specific interactions between each domain and the collagen triple helix.

In one crystal structure, ImpA binds a glycopeptide in an exosite located within its noncatalytic N-terminal domain (PF18650).[8] During our initial docking study, we observed that one of the accessory mucin-binding modules (Asn537-Leu650, PF03272) in SmE is also positioned to recognize additional sites in mucin substrates. At present, there is no experimentally determined structure of a PF03272 domain; thus, the precise details of the domain's structure and ligand recognition remain unknown. As such, we grafted larger segments of TIM-4 (*vide infra*) onto the docked glycopeptide to determine if this accessory domain can potentially bind the substrate. We observed that several of the larger TIM-4 substrates positioned glycans adjacent to a predicted binding pocket formed from two conserved segments in the mucin-binding module (Supplementary Figure 14, 15). While additional work is required to validate this initial finding, the result suggests that SmE could use its mucin-binding module to cooperatively bind mucin substrates and sterically occlude more globular O-glycoproteins. Such a model would explain SmE's observed preference for mucins over non-mucin O-glycoproteins like fetuin.

Supplementary References

1. Stavenhagen, K. *et al.* N- and *O* -glycosylation Analysis of Human C1-inhibitor Reveals Extensive Mucin-type *O* -Glycosylation. *Mol. Cell. Proteomics* **17**, 1225–1238 (2018).
2. Bock, S. C. *et al.* Human C.hivin.1 inhibitor: primary structure, cDNA cloning, and chromosomal localization. *Biochemistry* **25**, 4292–4301 (1986).
3. Pluvinage, B. *et al.* Architecturally complex *O* -glycopeptidases are customized for mucin recognition and hydrolysis. *Proc. Natl. Acad. Sci.* **118**, e2019220118 (2021).
4. Medley, B. J. *et al.* A previously uncharacterized O-glycopeptidase from Akkermansia muciniphila requires the Tn-antigen for cleavage of the peptide bond. *J. Biol. Chem.* **298**, 102439 (2022).
5. Pedram, K. *et al. Design of a mucin-selective protease for targeted degradation of cancer-associated mucins*. http://biorxiv.org/lookup/doi/10.1101/2022.05.20.492748 (2022) doi:10.1101/2022.05.20.492748.
6. Yu, A. C. Y., Worrall, L. J. & Strynadka, N. C. J. Structural Insight into the Bacterial Mucinase StcE Essential to Adhesion and Immune Evasion during Enterohemorrhagic E. coli Infection. *Structure* **20**, 707–717 (2012).
7. Manka, S. W. *et al.* Structural insights into triple-helical collagen cleavage by matrix metalloproteinase 1. *Proc. Natl. Acad. Sci.* **109**, 12461–12466 (2012).
8. Noach, I. & Boraston, A. B. Structural evidence for a proline-specific glycopeptide recognition domain in an O-glycopeptidase. *Glycobiology* **31**, 385–390 (2021).
9. Hollenhorst, M. A. *et al.* Comprehensive analysis of platelet glycoprotein Ibα ectodomain glycosylation. *J. Thromb. Haemost.* S1538783623000375 (2023) doi:10.1016/j.jtha.2023.01.009.
10. Lu, L., Riley, N. M., Shortreed, M. R., Bertozzi, C. R. & Smith, L. M. O-Pair Search with MetaMorpheus for O-glycopeptide characterization. *Nat. Methods* **17**, 1133–1138 (2020).
11. Guvench, O. *et al.* CHARMM Additive All-Atom Force Field for Carbohydrate Derivatives and Its Utility in Polysaccharide and Carbohydrate–Protein Modeling. *J. Chem. Theory Comput.* **7**, 3162–3180 (2011).
12. Rietz, T. A. *et al.* Fragment-Based Discovery of Small Molecules Bound to T-Cell Immunoglobulin and Mucin Domain-Containing Molecule 3 (TIM-3). *J. Med. Chem.* **64**, 14757–14772 (2021).

13. Raveh, B., London, N. & Schueler-Furman, O. Sub-angstrom modeling of complexes between flexible peptides and globular proteins: Sub-Angstrom Modeling of Flexible Peptides. *Proteins Struct. Funct. Bioinforma.* **78**, 2029–2040 (2010).

14. Jumper, J. *et al.* Highly accurate protein structure prediction with AlphaFold. *Nature* **596**, 583–589 (2021).

15. Varadi, M. *et al.* AlphaFold Protein Structure Database: massively expanding the structural coverage of protein-sequence space with high-accuracy models. *Nucleic Acids Res.* **50**, D439–D444 (2022).

16. Liu, N. *et al.* Crystal structures of human TIM members: Ebolavirus entry-enhancing receptors. *Chin. Sci. Bull.* **60**, 3438–3453 (2015).

17. Phillips, J. C. *et al.* Scalable molecular dynamics with NAMD. *J. Comput. Chem.* **26**, 1781–1802 (2005).

18. Olsson, M. H. M., Søndergaard, C. R., Rostkowski, M. & Jensen, J. H. PROPKA3: Consistent Treatment of Internal and Surface Residues in Empirical p K_a Predictions. *J. Chem. Theory Comput.* **7**, 525–537 (2011).

19. Jo, S., Song, K. C., Desaire, H., MacKerell, A. D. & Im, W. Glycan reader: Automated sugar identification and simulation preparation for carbohydrates and glycoproteins. *J. Comput. Chem.* **32**, 3135–3141 (2011).

20. Casares, D., Escribá, P. V. & Rosselló, C. A. Membrane Lipid Composition: Effect on Membrane and Organelle Structure, Function and Compartmentalization and Therapeutic Avenues. *Int. J. Mol. Sci.* **20**, 2167 (2019).

21. van Meer, G., Voelker, D. R. & Feigenson, G. W. Membrane lipids: where they are and how they behave. *Nat. Rev. Mol. Cell Biol.* **9**, 112–124 (2008).

22. Phillips, J. C. *et al.* Scalable molecular dynamics on CPU and GPU architectures with NAMD. *J. Chem. Phys.* **153**, 044130 (2020).

23. Guvench, O., Hatcher, E., Venable, R. M., Pastor, R. W. & MacKerell, A. D. CHARMM Additive All-Atom Force Field for Glycosidic Linkages between Hexopyranoses. *J. Chem. Theory Comput.* **5**, 2353–2370 (2009).

24. Huang, J. & MacKerell, A. D. CHARMM36 all-atom additive protein force field: Validation based on comparison to NMR data. *J. Comput. Chem.* **34**, 2135–2145 (2013).

25. Huang, J. *et al.* CHARMM36m: an improved force field for folded and intrinsically disordered proteins. *Nat. Methods* **14**, 71–73 (2017).

26. Klauda, J. B. *et al.* Update of the CHARMM All-Atom Additive Force Field for Lipids: Validation on Six Lipid Types. *J. Phys. Chem. B* **114**, 7830–7843 (2010).
27. Beglov, D. & Roux, B. Finite representation of an infinite bulk system: Solvent boundary potential for computer simulations. *J. Chem. Phys.* **100**, 9050–9063 (1994).
28. Han, K. *et al.* Graph–Theoretic Analysis of Monomethyl Phosphate Clustering in Ionic Solutions. *J. Phys. Chem. B* **122**, 1484–1494 (2018).
29. Venable, R. M., Luo, Y., Gawrisch, K., Roux, B. & Pastor, R. W. Simulations of Anionic Lipid Membranes: Development of Interaction-Specific Ion Parameters and Validation Using NMR Data. *J. Phys. Chem. B* **117**, 10183–10192 (2013).
30. Center, S. D. S. Triton Shared Computing Cluster. (2022) doi:10.57873/T34W2R.
31. Gowers, R. *et al.* MDAnalysis: A Python Package for the Rapid Analysis of Molecular Dynamics Simulations. in 98–105 (2016). doi:10.25080/Majora-629e541a-00e.
32. Michaud-Agrawal, N., Denning, E. J., Woolf, T. B. & Beckstein, O. MDAnalysis: A toolkit for the analysis of molecular dynamics simulations. *J. Comput. Chem.* **32**, 2319–2327 (2011).
33. Noach, I. *et al.* Recognition of protein-linked glycans as a determinant of peptidase activity. *Proc. Natl. Acad. Sci.* **114**, E679–E688 (2017).
34. Taleb, V. *et al.* Structural and mechanistic insights into the cleavage of clustered O-glycan patches-containing glycoproteins by mucinases of the human gut. *Nat. Commun.* **13**, 4324 (2022).
35. Shon, D. J., Fernandez, D., Riley, N. M., Ferracane, M. J. & Bertozzi, C. R. Structure-guided mutagenesis of a mucin-selective metalloprotease from Akkermansia muciniphila alters substrate preferences. *J. Biol. Chem.* **298**, 101917 (2022).
36. Pluvinage, B. *et al.* Architecturally complex *O* -glycopeptidases are customized for mucin recognition and hydrolysis. *Proc. Natl. Acad. Sci.* **118**, e2019220118 (2021).
37. Cerdà-Costa, N. & Xavier Gomis-Rüth, F. Architecture and function of metallopeptidase catalytic domains: Metallopeptidase Catalytic Domains. *Protein Sci.* **23**, 123–144 (2014).
38. Weiner, S. J. *et al.* A new force field for molecular mechanical simulation of nucleic acids and proteins. *J. Am. Chem. Soc.* **106**, 765–784 (1984).

39. Malaker, S. A. *et al.* The mucin-selective protease StcE enables molecular and functional analysis of human cancer-associated mucins. *Proc. Natl. Acad. Sci.* **116**, 7278–7287 (2019).

40. Jo, S. & Im, W. Glycan fragment database: a database of PDB-based glycan 3D structures. *Nucleic Acids Res.* **41**, D470–D474 (2012).

41. Pyburn, T. M. *et al.* A Structural Model for Binding of the Serine-Rich Repeat Adhesin GspB to Host Carbohydrate Receptors. *PLoS Pathog.* **7**, e1002112 (2011).

42. Somers, W. S., Tang, J., Shaw, G. D. & Camphausen, R. T. Insights into the Molecular Basis of Leukocyte Tethering and Rolling Revealed by Structures of P- and E-Selectin Bound to SLeX and PSGL-1. *Cell* **103**, 467–479 (2000).

43. Labute, P. LowModeMD—Implicit Low-Mode Velocity Filtering Applied to Conformational Search of Macrocycles and Protein Loops. *J. Chem. Inf. Model.* **50**, 792–800 (2010).

44. Smith, C. M., Li, A., Krishnamurthy, N. & Lemmon, M. A. Phosphatidylserine binding directly regulates TIM-3 function. *Biochem. J.* **478**, 3331–3349 (2021).

45. Itoh, A. *et al.* Optimization of the inter-domain structure of galectin-9 for recombinant production. *Glycobiology* **23**, 920–925 (2013).

46. Nishi, N. A note on expression and purification of recombinant galectins. Preprint at https://doi.org/10.32285/glycoforum.23A15 (2020).
