# Supplementary material for: Glycoproteomic landscape and structural dynamics of TIM family immune checkpoints enabled by mucinase SmE
Source: Nat Commun. 2023 Oct 4;14:6169. doi: 10.1038/s41467-023-41756-y (PMC10550946; doi:10.1038/s41467-023-41756-y)
Supplement: Supplementary file 3 — Description of Additional Supplementary files [file 41467_2023_41756_MOESM3_ESM.docx]

Description of Additional Supplementary Files for:

Glycoproteomic landscape and structural dynamics of TIM family immune checkpoints enabled by mucinase SmE

File Name: Supplementary Data 1

Description: C1-INH glycopeptides identified

File Name: Supplementary Data 2

Description: Gp1b-alpha glycopeptides identified

File Name: Supplementary Data 3

Description: TIM-1 glycopeptides identified

File Name: Supplementary Data 4

Description: TIM-3 glycopeptides identified

File Name: Supplementary Data 5

Description: TIM-4 glycopeptides identified

File Name: Supplementary Data 6

Description: Fetuin glycopeptides identified

File Name: Supplementary Data 7

Description: Quantification of glycan structures on TIM-1

File Name: Supplementary Data 8

Description: Quantification of glycan structures on TIM-4
